# Supplementary material for: Massively parallel identification of sequence motifs triggering ribosome-associated mRNA quality control
Source: bioRxiv. 2023 Sep 27:2023.09.27.559793. Preprint. [Version 1] doi: 10.1101/2023.09.27.559793 (PMC10557687; doi:10.1101/2023.09.27.559793)
Supplement: Supplement 1 [file NIHPP2023.09.27.559793v1-supplement-1.pdf]

bioRxiv preprint doi: https://doi.org/10.1101/2023.09.27.559793; this version posted September 27, 2023. The copyright holder for this preprint (which was not certified by peer review) is the author/funder, who has granted bioRxiv a license to display the preprint in perpetuity. It is made available under aCC-BY 4.0 International license.

Supplementary Figures

Figure S1

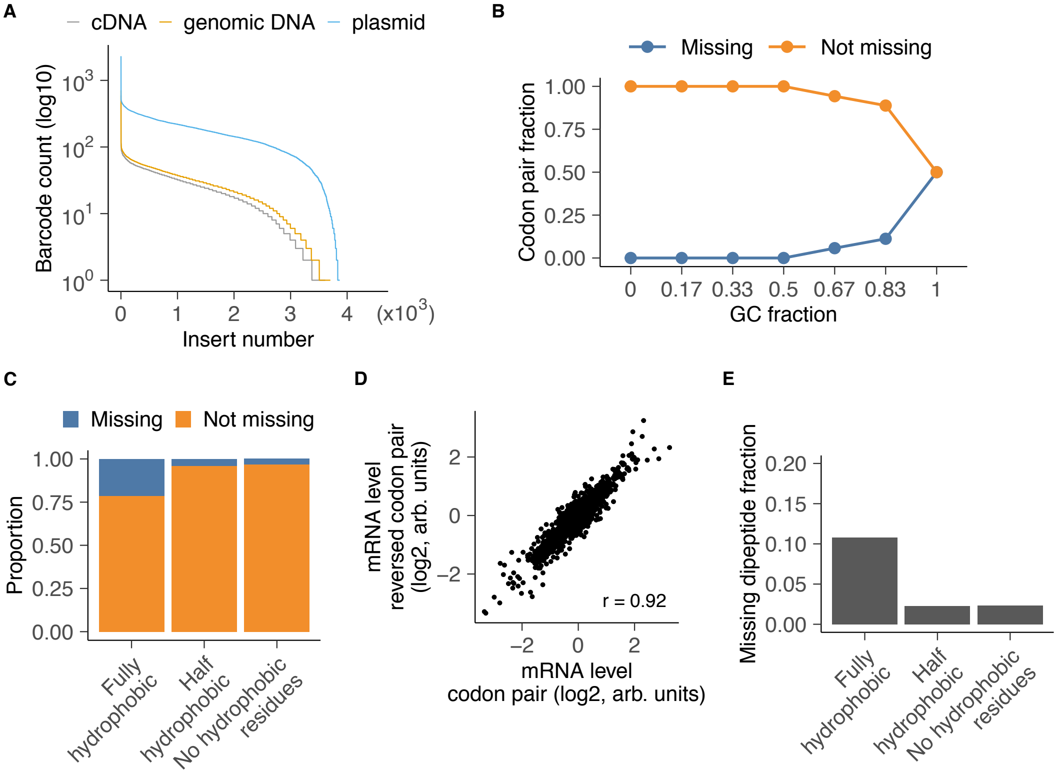

Plasmid and yeast codon pair library alignment statistics

(A) Distribution of barcodes per codon pair insert for the plasmid, cDNA, and genomic DNA libraries. **(B)** Proportion of missing codon pair inserts in the plasmid library by GC content. **(C)** Proportion of missing codon pair inserts in wild-type yeast grouped by hydrophobicity. **(D)** mRNA level of reporters for each codon pair compared to its reversed codon pair. Stop codon-containing pairs and pairs where the codon and reversed codon are the same are excluded. *r* indicates Pearson correlation coefficient. **(E)** Proportion of missing codon pair inserts grouped by hydrophobicity for the 139 inserts that are missing in all three strains.

bioRxiv preprint doi: https://doi.org/10.1101/2023.09.27.559793; this version posted September 27, 2023. The copyright holder for this preprint (which was not certified by peer review) is the author/funder, who has granted bioRxiv a license to display the preprint in perpetuity. It is made available under aCC-BY 4.0 International license.

Figure S2

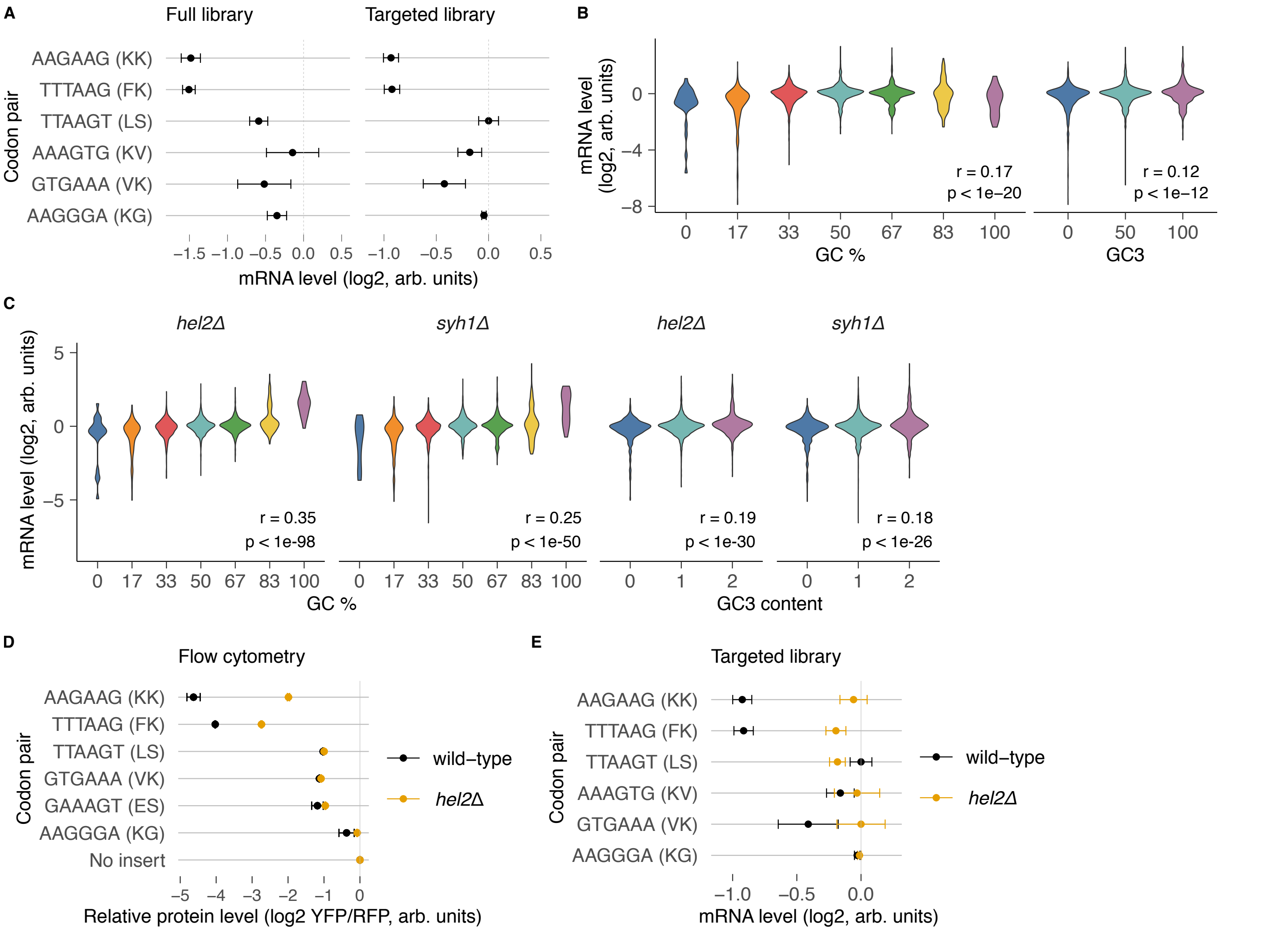

(A) Small-scale validation of codon pair screen. The mRNA levels for individually cloned codon pair inserts (as described in Fig. 2C, D) are plotted alongside mRNA levels for the same codon pairs taken from the full library. Error bars are calculated as in Fig. 3C. mRNA levels for the small-scale library are normalized to the maximum value and mRNA levels for the full library are normalized to the median value. **(B)** mRNA levels of codon pair inserts as a function of their GC content (left) or GC3 content (right) in wild-type cells. Pearson correlation coefficient r and p-value p are shown for GC content and GC3 content (two-sided t-test). **(C)** Same as in B, but for *hel2Δ* and *syh1Δ* cells. **(D)** Effect of individually cloned codon pair inserts on peptide expression in *hel2Δ* cells compared to wild-type. Peptide expression is quantified as in Fig. 2D. **(E)** mRNA level of individually cloned codon pair inserts in *hel2Δ* cells compared to wild-type. mRNA levels and error bars are calculated as in Fig. 3C, except with maximum-normalization.

bioRxiv preprint doi: https://doi.org/10.1101/2023.09.27.559793; this version posted September 27, 2023. The copyright holder for this preprint (which was not certified by peer review) is the author/funder, who has granted bioRxiv a license to display the preprint in perpetuity. It is made available under aCC-BY 4.0 International license.

Figure S3

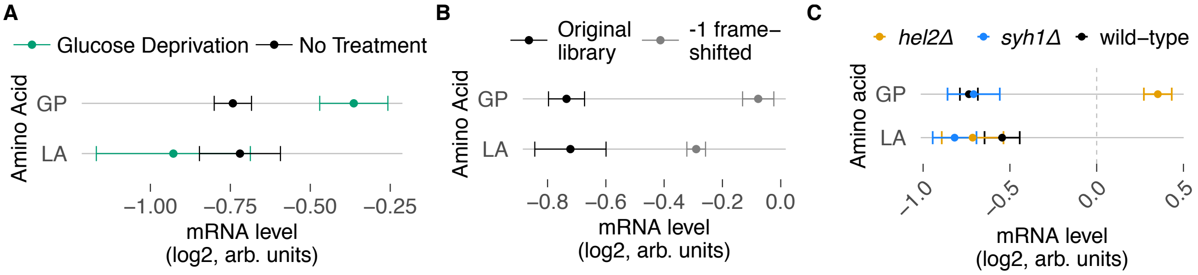

(A) mRNA levels for reciprocal dipeptide repeats not included in Fig. 3C. **(B)** mRNA levels for reciprocal dipeptide repeats not included in Fig. 3F. **(C)** mRNA levels for reciprocal dipeptide repeats not included in Fig. 4E.

bioRxiv preprint doi: https://doi.org/10.1101/2023.09.27.559793; this version posted September 27, 2023. The copyright holder for this preprint (which was not certified by peer review) is the author/funder, who has granted bioRxiv a license to display the preprint in perpetuity. It is made available under aCC-BY 4.0 International license.

Supplementary Tables

Table S1

List of plasmids used for this study

Plasmid	Genotype	Figure	Source
pHPSC16	pUC-HO3-LEU2-pTDH3-mKate2-tCYC1	parent	[20]
pHPSC417	pAG306-GPD-3xFLAG-PGK1-YFP	parent	[20]
pHPSC1120	pAG306-pGPD-3xFLAG-PGK1-no-insert-R1-T7-YFP	parent, 2	This work
pHPSC1114	pAG306-pGPD-3xFLAG-PGK1_-1-BamHI-R1-T7-YFP	parent	This work
pHPHS1142	pAG306-pGPD-3xFLAG-PGK1-8×dicodon-endofragments-24ntbarc-R1-T7-YFP	1, 2, 3, 4, 6	This work
pHPSC1117	pAG306-pGPD-3xFLAG-PGK1_-1-8×dicodon-endofrag-24ntbarc-R1-T7-YFP	3	This work
pHPSC1163	pAG306-pGPD-3xFLAG-PGK1-FK_8dms-R1-T7-YFP	5	This work
pHPSC1160	pAG306_pGPD-3xFLAG-PGK1-8×_minipool-24VNN-R1-T7-YFP	Supplemental 2	This work
pHPSC1144	pAG306-pGPD-3xFLAG-PGK1-VK_GTGAAA-R1-T7-YFP	2	This work
pHPSC1145	pAG306-pGPD-3xFLAG-PGK1-FK_TTTAAG-R1-T7-YFP	2	This work
pHPSC1146	pAG306-pGPD-3xFLAG-PGK1-ES_GAAAGT-R1-T7-YFP	2	This work
pHPSC1147	pAG306-pGPD-3xFLAG-PGK1-LS_TTAAGT-R1-T7-YFP	2	This work
pHPSC1149	pAG306-pGPD-3xFLAG-PGK1-KK_AAGAAG-R1-T7-YFP	2	This work
pHPSC1150	pAG306-pGPD-3xFLAG-PGK1-KG_AAGGGA-R1-T7-YFP	2	This work
pHPSC1159-sc2	pAG306_pGPD_PGK1_spikein2_24ntbarc_R1_YFP	5	This work
pHPSC1159-sc5	pAG306_pGPD_PGK1_spikein5_24ntbarc_R1_YFP	5	This work

bioRxiv preprint doi: https://doi.org/10.1101/2023.09.27.559793; this version posted September 27, 2023. The copyright holder for this preprint (which was not certified by peer review) is the author/funder, who has granted bioRxiv a license to display the preprint in perpetuity. It is made available under aCC-BY 4.0 International license.

Table S2

List of *S. cerevisiae* strains used for this study

Strain	Genotype, integrated plasmid	Figure	Source
BY4741	S288C, MATa HIS3Δ1 LEU2Δ0 MET15Δ0 URA3Δ0	Parent	Thermo Fisher
scHP15	BY4741, pHPSC16	Parent	[20]
scHP520	scHP15, HEL2::NAT	Parent	[20]
scHP1408	BY4741, HEL2::KanMX	Parent	This work
scKC192	BY4741, SYH1::KanMX	4	This work
scKC190	BY4741, pHPSC1159-sc2	5	This work
scKC191	BY4741, pHPSC1159-sc5	5	This work

bioRxiv preprint doi: https://doi.org/10.1101/2023.09.27.559793; this version posted September 27, 2023. The copyright holder for this preprint (which was not certified by peer review) is the author/funder, who has granted bioRxiv a license to display the preprint in perpetuity. It is made available under aCC-BY 4.0 International license.

Table S3

List of oligos used for this study

Oligo number	Oligo name	Oligo sequence
oKC97	HA	GACGTCCCGGACTACGCG
oHP531	24nt_VNN_barcode_read1	cacgacgctcttccgatctNNBNNBNNBNNBNNBNNBNNBNNBGAACAATTCTTCACCCT
oKC129	VK_GTGAAA_rev	TCTCGTAGCATCACTCCGTC
oKC130	VK_GTGAAA_fwd	GGGGTTAGCTTTCGCAATTC
oKC131	FK_TTTAAG_rev	TGGAGTTGTGCTGGCTTCGT
oKC132	FK_TTTAAG_fwd	TGTACATAAGAGGTCGTCAC
oKC133	ES_GAAAGT_rev	AGAGCATTTGGGGCTGCCCG
oKC134	ES_GAAAGT_fwd	CCAGTCGTCAGCCAATAGTG
oKC135	LS_TTAAGT_rev	CACAAGCGAGGGGAGGGTTA
oKC136	LS_TTAAGT_fwd	TGAGGAGCGCTAGGAGCAGT
oKC139	KK_AAGAAG_rev	GCTGCAACAATGTATCTGAG
oKC140	KK_AAGAAG_fwd	CAGTGTGAGAGTCCTGGTAG
oKC141	KG_AAGGGA_rev	GTCGAATACGTATGTGGGGA
oKC142	KV_AAGGGA_fwd	GGCTCCGTTGTTGATGCCTT
oKC123	R1_oAS385_rev	CACGACGCTCTTCCGATCTTTCTTCACCCTTAGACAT
oKC148	24nt_VNN_scoverhang	cacgacgctcttccgatctNNBNNBNNBNNBNNBNNBNNBNNBTTCTTCACCCTTAGACA
oHP534	read2_scoverhang_fwd	gtgactggagttcagacgtgtgctcttccgatctATGTCTAAGGGTGAAGAATTGTTC
oHP528	PGK1_-1_HA_fwd	ggtgttgctttcttatccgaaaagaaaTGTACCCGTACGACGTCCCGGACTACGC
oHP529	YFP_T7_rev	gattggaacaacaccagtgaacagctcctctcccttGTAATACGACTCACTATAG
oHP530	HA_illumina_fwd	TACCCGTACGACGTCCCGGACTACGCGggatccatcagatcggaagagcgtcg
oHP558	PGK1_HA_fwd	gtgttgctttcttatccgaaaagaaaTACCCGTACGACGTCCC
oHP532	HA_sc_overhang_fwd	TGTACCCGTACGACGTCCC
oKC225	scoverhang_R1_ev	cacgacgctcttccgatctGAACAATTCTTCACCCTTAGACAT
oAS135	PCR_P7_R2_ix_24	CAAGCAGAAGACGGCATACGAGATGGTAGCGTGACTGGAGTTCAGACGTGTGCTC
oAS134	PCR_P7_R2_ix_23	CAAGCAGAAGACGGCATACGAGATGAGTGGGTGACTGGAGTTCAGACGTGTGCTC
oAS133	PCR_P7_R2_ix_22	CAAGCAGAAGACGGCATACGAGATCGTACGGTGACTGGAGTTCAGACGTGTGCTC
oAS132	PCR_P7_R2_ix_21	CAAGCAGAAGACGGCATACGAGATGTTTCGGTGACTGGAGTTCAGACGTGTGCTC
oAS131	PCR_P7_R2_ix_20	CAAGCAGAAGACGGCATACGAGATGTGGCCGTGACTGGAGTTCAGACGTGTGCTC
oAS130	PCR_P7_R2_ix_19	CAAGCAGAAGACGGCATACGAGATGTGAAAGTGACTGGAGTTCAGACGTGTGCTC
oAS129	PCR_P7_R2_ix_18	CAAGCAGAAGACGGCATACGAGATGTCCGCGTGACTGGAGTTCAGACGTGTGCTC
oAS128	PCR_P7_R2_ix_17	CAAGCAGAAGACGGCATACGAGATGTAGAGGTGACTGGAGTTCAGACGTGTGCTC
oAS127	PCR_P7_R2_ix_16	CAAGCAGAAGACGGCATACGAGATCCGTCCGTGACTGGAGTTCAGACGTGTGCTC
oAS126	PCR_P7_R2_ix_15	CAAGCAGAAGACGGCATACGAGATATGTCAGTGACTGGAGTTCAGACGTGTGCTC
oAS125	PCR_P7_R2_ix_14	CAAGCAGAAGACGGCATACGAGATAGTTCCGTGACTGGAGTTCAGACGTGTGCTC
oAS124	PCR_P7_R2_ix_13	CAAGCAGAAGACGGCATACGAGATAGTCAAGTGACTGGAGTTCAGACGTGTGCTC
oAS123	PCR_P7_R2_ix_12	CAAGCAGAAGACGGCATACGAGATCTTGTAGTGACTGGAGTTCAGACGTGTGCTC
oAS122	PCR_P7_R2_ix_11	CAAGCAGAAGACGGCATACGAGATGGCTACGTGACTGGAGTTCAGACGTGTGCTC
oAS121	PCR_P7_R2_ix_10	CAAGCAGAAGACGGCATACGAGATTAGCTTGTGACTGGAGTTCAGACGTGTGCTC
oAS120	PCR_P7_R2_ix_9	CAAGCAGAAGACGGCATACGAGATGATCAGGTGACTGGAGTTCAGACGTGTGCTC
oAS119	PCR_P7_R2_ix_8	CAAGCAGAAGACGGCATACGAGATACTTGAGTGACTGGAGTTCAGACGTGTGCTC
oAS118	PCR_P7_R2_ix_7	CAAGCAGAAGACGGCATACGAGATCAGATCGTGACTGGAGTTCAGACGTGTGCTC
oAS117	PCR_P7_R2_ix_6	CAAGCAGAAGACGGCATACGAGATGCCAATGTGACTGGAGTTCAGACGTGTGCTC
oAS116	PCR_P7_R2_ix_5	CAAGCAGAAGACGGCATACGAGATACAGTGGTGACTGGAGTTCAGACGTGTGCTC
oAS115	PCR_P7_R2_ix_4	CAAGCAGAAGACGGCATACGAGATTGACCAGTGACTGGAGTTCAGACGTGTGCTC
oAS114	PCR_P7_R2_ix_3	CAAGCAGAAGACGGCATACGAGATTTAGGCGTGACTGGAGTTCAGACGTGTGCTC
oAS113	PCR_P7_R2_ix_2	CAAGCAGAAGACGGCATACGAGATCGATGTGTGACTGGAGTTCAGACGTGTGCTC
oAS112	PCR_P7_R2_ix_1	CAAGCAGAAGACGGCATACGAGATATCACGGTGACTGGAGTTCAGACGTGTGCTC

bioRxiv preprint doi: https://doi.org/10.1101/2023.09.27.559793; this version posted September 27, 2023. The copyright holder for this preprint (which was not certified by peer review) is the author/funder, who has granted bioRxiv a license to display the preprint in perpetuity. It is made available under aCC-BY 4.0 International license.

Oligo number	Oligo name	Oligo sequence
oAS111	PCR_common_P5_R1	AATGATACGGCGACCACCGAGATCTACACTCTTTCCCTACACGACGCTC
oHP290	PCR_P7_R2_ix_34	CAAGCAGAAGACGGCATACGAGATCATGGCGTGACTGGAGTTCAGACGTGTGCTC
oHP289	PCR_P7_R2_ix_33	CAAGCAGAAGACGGCATACGAGATCAGGCGGTGACTGGAGTTCAGACGTGTGCTC
oHP288	PCR_P7_R2_ix_32	CAAGCAGAAGACGGCATACGAGATCACTCAGTGACTGGAGTTCAGACGTGTGCTC
oHP287	PCR_P7_R2_ix_31	CAAGCAGAAGACGGCATACGAGATCACGATGTGACTGGAGTTCAGACGTGTGCTC
oHP286	PCR_P7_R2_ix_30	CAAGCAGAAGACGGCATACGAGATCACCGGGTGACTGGAGTTCAGACGTGTGCTC
oHP285	PCR_P7_R2_ix_29	CAAGCAGAAGACGGCATACGAGATCAACTAGTGACTGGAGTTCAGACGTGTGCTC
oHP284	PCR_P7_R2_ix_28	CAAGCAGAAGACGGCATACGAGATCAAAAGGTGACTGGAGTTCAGACGTGTGCTC
oHP283	PCR_P7_R2_ix_27	CAAGCAGAAGACGGCATACGAGATATTCCTGTGACTGGAGTTCAGACGTGTGCTC
oHP282	PCR_P7_R2_ix_26	CAAGCAGAAGACGGCATACGAGATATGAGCGTGACTGGAGTTCAGACGTGTGCTC
oHP281	PCR_P7_R2_ix_25	CAAGCAGAAGACGGCATACGAGATACTGATGTGACTGGAGTTCAGACGTGTGCTC
oPN776	P5_grafting_R	aatgatacggcgaccaccgagatctacac
oKC235	R1_homology_umi_P5_RT	gcgaccaccgagatctACACNNNNNNNACACTCTTTCCCTAcacgacgctcttccgatct
oKC254	P7_ix_16_HA	caagcagaagacggcatacgagatCCGTCCCCGTACGACGTCCCGGACTACGCG
oKC253	P7_ix_15_HA	caagcagaagacggcatacgagatATGTCACCGTACGACGTCCCGGACTACGCG
oKC252	P7_ix_14_HA	caagcagaagacggcatacgagatAGTTCCCCGTACGACGTCCCGGACTACGCG
oKC251	P7_ix_13_HA	caagcagaagacggcatacgagatAGTCAACCGTACGACGTCCCGGACTACGCG
oKC250	P7_ix_17_HA	caagcagaagacggcatacgagatGTAGAGCCGTACGACGTCCCGGACTACGCG
oKC249	P7_ix_29_HA	caagcagaagacggcatacgagatCAACTACCGTACGACGTCCCGGACTACGCG
oKC248	P7_ix_28_HA	caagcagaagacggcatacgagatCAAAAGCCGTACGACGTCCCGGACTACGCG
oKC247	P7_ix_27_HA	caagcagaagacggcatacgagatATTCCTCCGTACGACGTCCCGGACTACGCG
oKC246	P7_ix_26_HA	caagcagaagacggcatacgagatATGAGCCCGTACGACGTCCCGGACTACGCG
oKC245	P7_ix_25_HA	caagcagaagacggcatacgagatACTGATCCGTACGACGTCCCGGACTACGCG
oKC244	P7_ix_24_HA	caagcagaagacggcatacgagatGGTAGCCCGTACGACGTCCCGGACTACGCG
oKC243	P7_ix_23_HA	caagcagaagacggcatacgagatGAGTGGCCGTACGACGTCCCGGACTACGCG
oKC242	P7_ix_22_HA	caagcagaagacggcatacgagatCGTACGCCGTACGACGTCCCGGACTACGCG
oKC241	P7_ix_21_HA	caagcagaagacggcatacgagatGTTTCGCCGTACGACGTCCCGGACTACGCG
oKC240	P7_ix_20_HA	caagcagaagacggcatacgagatGTGGCCCCGTACGACGTCCCGGACTACGCG
oKC239	P7_ix_19_HA	caagcagaagacggcatacgagatGTGAAACCGTACGACGTCCCGGACTACGCG
oKC238	P7_ix_18_HA	CAAGCAGAAGACGGCATACGAGATGTCCGCCCGTACGACGTCCCGGACTACGCG
oKC232	P7_ix_32_HA	caagcagaagacggcatacgagatCACTCACCGTACGACGTCCCGGACTACGCG
oKC231	P7_ix_31_HA	caagcagaagacggcatacgagatCACGATCCGTACGACGTCCCGGACTACGCG
oKC230	P7_ix_30_HA	caagcagaagacggcatacgagatCACCGGCCGTACGACGTCCCGGACTACGCG
oKC234	P7_ix_34_HA	caagcagaagacggcatacgagatCATGGCCCGTACGACGTCCCGGACTACGCG
oKC233	P7_ix_33_HA	caagcagaagacggcatacgagatCAGGCGCCGTACGACGTCCCGGACTACGCG
oAS385	Pool 1	TGTACCCGTACGACGTCCCGGACTACGCGNNNNNNNNNNNNNNNNNNNNNNNN<br>NNNNNNNNNNNNNNNNNNNNNNNNNATGTCTAAGGGTGAAGAATTGTTC
oKC224	Pool 2	GACGTCCCGGACTACGCGNNNNNNNNNNNNNNNNNNNNNNNN<br>NNNNNNNNNNNNNNNNNNNNNNNNNATGTCTAAGGGTGAAGAATTGTTC
oPB354	Standard Read 1 sequencing primer	acactctttccctacacgacgctcttccgatct
oRB80	Standard Read 2 sequencing primer	GTGACTGGAGTTCAGACGTGTGCTCTTCCGATCT
oAS1638	Custom Read 2 sequencing primer	cgtgtgctcttccgatctATGTCTAAGGGTG
oAS1637	Custom Read 2 sequencing primer	atctCCGTACGACGTCCCGGACTACGCG
oKC236	Custom Read 2 sequencing primer	CCGTACGACGTCCCGGACTACGCG

bioRxiv preprint doi: https://doi.org/10.1101/2023.09.27.559793; this version posted September 27, 2023. The copyright holder for this preprint (which was not certified by peer review) is the author/funder, who has granted bioRxiv a license to display the preprint in perpetuity. It is made available under aCC-BY 4.0 International license.

Oligo number	Oligo name	Oligo sequence
oKC256	Custom i7 index sequencing primer	CGCGTAGTCCGGGACGTCGTACGG
oPN705	Standard i7 index sequencing primer	AGATCGGAAGAGCACACGTCTGAACTCCAGTCAC
oKC255	Standard i5 index sequencing primer	agatcggaagagcgtcgtgTAGGGAAAGAGTGT
